# Association of eight urinary metals and trouble sleeping: A cross-sectional study

**DOI:** 10.1097/MD.0000000000050551

**Published:** 2026-09-04

**Authors:** Qiong Ma, Jian-Zheng Liu, Xiu-E. Shi, Li-Li Li, Xiao-Wei Feng, Xiang Fan, Mi Fu, Jian-Min Lv

**Affiliations:** a Health Department, Northwest Women’s and Children’s Hospital, Xi’an, Shaanxi, P.R. China; b Department of Cardiology, Xijing Hospital, Xi’an, Shaanxi, P.R. China; c Rehabilitation Science Institute, Shaanxi Provincial Rehabilitation Hospital, Xi’an, Shaanxi, P.R. China.

**Keywords:** cross-sectional study, NHANES (National Health and Nutrition Examination Survey), trouble sleeping, urine metals

## Abstract

While toxic heavy metals have been linked to sleep disorders, the role of less-studied metals remains unclear. We aimed to explore the associations of 8 urinary metals with trouble sleeping and to identify potential gender-specific patterns. This study included 10,012 adults from the National Health and Nutrition Examination Survey 2005–2018. Urinary concentrations of tungsten, arsenic, thallium, cesium, antimony, molybdenum, cobalt, and cadmium were measured. Trouble sleeping was self-reported. We employed weighted logistic regression, restricted cubic spline models, and weighted quantile sum regression to evaluate single and mixed-metal effects. Among participants, 50.2% were male and 25.3% reported trouble sleeping. Higher urinary tungsten levels were associated with increased likelihood of trouble sleeping in males (odds ratios: 1.28–1.30). Arsenic, thallium, and cesium showed inverse associations, with notable gender differences. Weighted quantile sum analysis supported the positive association of tungsten and the inverse associations of arsenic and cesium. No significant associations were found for antimony, molybdenum, cobalt, or cadmium. Urinary tungsten is cross-sectionally associated with trouble sleeping in males, while arsenic, thallium, and cesium show inverse associations, with gender-specific patterns. Further research using validated tools is warranted to validate these associations, elucidate the underlying biological mechanisms, and highlight the significance of assessing combined metal exposures in relation to sleep health.

## 1. Introduction

The prevalence of sleep disturbances, including insomnia, restless legs syndrome, narcolepsy and sleep apnea, has been steadily increasing, affecting one-third of the US adults.^[1–3]^ These disorders not only diminish quality of life but also present significant public health challenges due to their associations with various comorbidities, such as cardiovascular diseases (CVD), metabolic disorders, and mental health issues.^[1–3]^

Recent research has increasingly focused on the environmental determinants of sleep health, particularly the role of heavy metals and trace elements, which are typically ingested into the human body through air, food, water, and occupational exposure.^[4–7]^ Heavy metals like lead, mercury, cadmium, and arsenic are known to induce oxidative stress and disrupt biological processes.^[8,9]^ Therefore, previous studies have paid attention to these toxic heavy metals and their associations with sleep disorders. However, the findings have often been based on relatively small sample sizes and yielded inconsistent results. For example, some studies based on general populations have reported significant association between cadmium exposure and various sleep disturbances,^[10,11]^ but others have found no association between blood cadmium levels and sleep duration or restless legs syndrome.^[12,13]^ Similarly, the association between arsenic exposure and sleep disturbances has also been mixed.^[13,14]^

In addition to these well-studied metals, other heavy metals and trace elements may also influence sleep health. However, research on these metals was limited, and existing studies were also constrained by small sample sizes and inconsistent findings. For instance, one study linked thallium exposure, commonly found near industrial areas, to various sleep-related issues,^[15]^ but another study based on general population found no significant association between thallium and sleep disorders.^[13]^ Other metals, such as cobalt, molybdenum, and cesium, have similarly been under-studied.^[13,16,17]^ Furthermore, the correlation between antimony exposure and sleep disorders also remained inconclusive,^[18,19]^ highlighting the need for further investigation.

Notably, emerging evidence from middle-income countries has also begun to inform this field, though findings remain preliminary. Yufu et al reported that plasma zinc, magnesium, and their ratios with copper and selenium were associated with reduced sleep disorder risk in a Chinese population using a mixture-based approach.^[8]^ Similarly, Wang et al found that urinary molybdenum, strontium, and magnesium were negatively associated with poor sleep quality in 3957 Chinese older adults, with the overall metal mixture also showing an inverse relationship.^[20]^

Overall, despite emerging evidences, research on associations between metal exposures and sleep disorders remains fragmented and inconsistent. Most studies have focused on a limited set of toxic heavy metals while neglecting other potentially impactful metals. Moreover, these studies differ widely in their focus, populations, and methodologies, leading to variable findings.

Our study aims to address these gaps by broadening the scope to include a wider range of metals that have been less studied in the context of sleep health-specifically tungsten, cesium, antimony, molybdenum, and cobalt-alongside the more frequently investigated arsenic, thallium, and cadmium. Utilizing data from the National Health and Nutrition Examination Survey (NHANES) from 2005 to 2018, we provide a comprehensive analysis of the association between concentrations of 8 urinary metals (tungsten, arsenic, thallium, cesium, antimony, molybdenum, cobalt, and cadmium) and trouble sleeping over a substantial period. We believe that this extensive dataset allows for a more accurate and generalizable understanding of the potential impacts of urinary metal concentrations on risk of trouble sleeping.

## 2. Methods

### 2.1. Data source and study population

NHANES is a cross-sectional analysis targeting U.S. citizens conducted by the National Center for Health Statistics, Hyattsville, MD, USA. This survey employs a complex, stratified, multistage probability cluster design. Briefly, primary sampling units (counties) are selected first, followed by segments within those counties, then households, and finally individual participants. This design ensures a nationally representative sample of the U.S. civilian noninstitutionalized population. Detailed information regarding NHANES can be found on its official website: https://www.cdc.gov/nchs/nhanes/. NHANES was approved by the Ethics Review Board of the National Center for Health Statistics and adhered to the ethical standards outlined in the Declaration of Helsinki and its subsequent amendments. In addition, each participant provided written informed consent voluntarily. This study is a secondary analysis of de-identified, publicly available NHANES data, and because we used only anonymized public-use data, additional ethics approval was not required.

**Figure 1.**
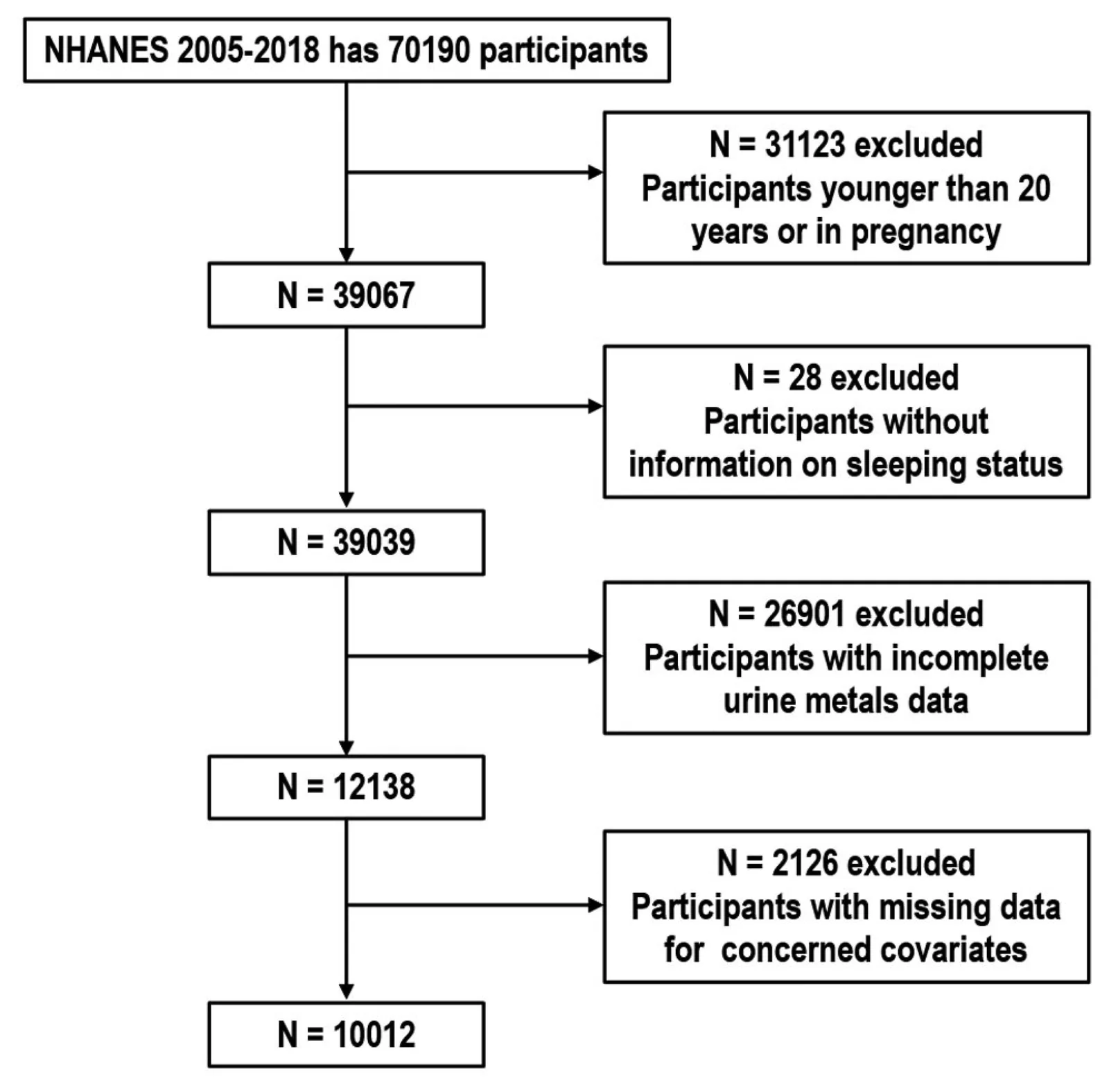
Flowchart of participant selection. NHANES = National Health and Nutrition Examination Survey.

In this study, data from 7 NHANES cycles spanning from 2005 to 2018 were involved. The demographics, examination results, questionnaire data, and laboratory data of the participants were extracted. A total of 10,012 Individuals who met all of the following conditions were included: with an age ≥ 20; not pregnant; with information on sleeping status; were measured for the urine metals; with complete data on all concerned covariates. The flowchart of participant selection is provided in Figure 1.

### 2.2. Trouble sleeping measurement

In NHANES, a series of questions related to sleep status were asked, which were all recorded in the “Sleep Disorders (SLQ)” questionnaire dataset. In this study, the variable SLQ050 (ever told a doctor or other health professional that you have trouble sleeping) was used to determine whether a participant has trouble sleeping, with the answer “yes” indicating the presence of trouble sleeping, and “no” indicating the absence of trouble sleeping.^[21]^ This self-reported item reflects perceived sleep disturbance, which is distinct from a clinically diagnosed sleep disorder, but was chosen because it is available across all NHANES cycles and has been widely used in population‑based research. We restricted our analysis to adults aged ≥ 20 years to minimize confounding by age-related physiological and lifestyle factors that differ substantially between children/adolescents and adults. Trouble sleeping was used as the outcome variable in this study.

### 2.3. Urine metals assessment

Urine specimens were processed, stored at −20°C, and shipped to the Division of Environmental Health Laboratory Sciences, National Center for Environmental Health, Centers for Disease Control and Prevention (Atlanta, GA, USA) for analysis. Concentrations of 8 urinary metals – tungsten, arsenic, thallium, cesium, antimony, molybdenum, cobalt, and cadmium – were measured using inductively coupled plasma‑mass spectrometry method. Values below the lower limit of detection were substituted with the lower limit of detection divided by the square root of 2, as recommended by NHANES. In order to eliminate detection errors caused by factors such as urine dilution, we used urine creatinine to correct the concentrations of each urine metal. The corrected metal concentration unit is “μg/g creatinine.”

### 2.4. Covariates

Potential covariates were considered in this study according to previous studies, which were: socio-demographic characteristics including age, gender, race/ethnicity, education level, poverty income ratio (PIR) and marital status; behavioral characteristics including smoking status and caffeine intake; and health factors including body mass index (BMI), diabetes mellitus (DM), CVD, and hypertension.

Age was categorized as 20 to <40, 40 to <60, and ≥60 years. Race/ethnicity was categorized into the following 5 groups: Mexican American, other Hispanic, non-Hispanic White, non-Hispanic Black, and other race-including multiracial. Education level was classified as <9th grade, 9 to 11th grade (includes 12th grade with no diploma), High school graduate/GED or equivalent, Some college or AA degree, and College graduate or above. PIR was represented as a continuous variable. Marital status was categorized into 3 groups: married/living with partner, never married, and widowed/divorced/separated. For smoking status, participants were divided into never-smokers (individuals who never smoked or smoked < 100 cigarettes in life) and smokers (having smoked ≥ 100 cigarettes in life). Caffeine intake was estimated using the DR1ICAFF variable from the NHANES dataset, and categorized into 3 groups based on tertile cutoffs (≤ 42, 43–164, and ≥165 mg/d). BMI was categorized into normal (< 25.0 kg/m^2^), overweight (25.0 to <30.0 kg/m^2^), and obese (≥ 30.0 kg/m^2^).

### 2.5. Statistical analysis

Since NHANES survey employed a series of complex sampling designs, appropriate sample weights were applied to account for oversampling and nonresponse in order to obtain nationally representative results. Weighted analyses were performed following the guidelines of the NHANES.

Categorical variables were expressed as numbers and percentages, and chi-squared tests were employed to compare the distribution between trouble sleeping and no trouble sleeping populations. Continuous variables were presented as median (Lower Quartile and Upper Quartile), and Wilcoxon rank-sum tests were employed to compare the difference between trouble sleeping and no trouble sleeping.

Urinary levels of 8 metals were divided into quartiles on the basis of their distributions in the current population, using the first quartile as the reference. The first, second, third and fourth quartiles are represented by Q1, Q2, Q3, and Q4, respectively. Weighted univariate logistic regression model (crude model) and multivariable logistic regression models (adjusted model) were employed to calculate the odds ratio (OR) and 95% confidence interval (95% CI) for the association between levels of 8 urinary metals and trouble sleeping. Adjusted model was adjusted for age, gender, race/ethnicity, education level, PIR, marital status, smoking status, caffeine intake, BMI, DM, CVD, and hypertension. To further determine the potential gender difference, subgroup analysis based on gender was performed.

Restricted cubic spline (RCS) regression with 3 knots (10th, 50th, and 90th percentiles) was applied to examine the nonlinearity of the association between urinary metals and trouble sleeping. In this analysis, urinary metals were performed logarithmic transformation. RCS regression was adjusted for age, gender, race/ethnicity, education level, PIR, marital status, smoking status, caffeine intake, BMI, DM, CVD, and hypertension. In addition, we also assessed effect modification by gender using stratification.

To examine the relationship between urinary metal mixtures and trouble sleeping, weighted quantile sum (WQS) regression was employed. The model converts urinary metal concentrations into quartiles and calculates a weighted index to show changes in sleep outcomes per quartile shift. It uses the bootstrap method to assign weights to each metal. The weights’ magnitude shows each metal’s contribution to the WQS index, with higher values indicating a more significant impact. All metals in the WQS model must be assumed to correlate in the same direction with the dependent variable. So, we built models for each metal in positive and negative directions to check for associations. The analysis was performed using the “gWQS” R package. To avoid overfitting and provide validation power, data were tested and validated using 40% and 60% stratified random samples, with 1000 bootstrap iterations.

To assess the robustness of the findings, a sensitivity analysis focusing on the influence of extreme values was conducted. Urinary metal concentrations were log-transformed, and outliers were defined as values greater than the mean +3 standard deviations or less than the mean −3 standard deviations. These outliers were winsorized by replacing them with the 99th percentile value of the respective metal distribution. Logistic regression and WQS models were reestimated using the adjusted dataset to test the stability of the results.

Statistical analysis was performed using R statistical software version 4.3 (R Project for Statistical Computing). A two-sided *P* value < .05 was considered statistically significant.

## 3. Results

### 3.1. Baseline characteristics of the participants

Among the 10,012 participants included from the NHANES 2005–2018, 5026 (50.2%) were male and 2535 (25.3%) had trouble sleeping. Detailed information about the baseline characteristics of all participants grouped by sleep status was illustrated in Table 1. Overall, age, gender, race/ethnicity, education level, marital status, smoking status, caffeine intake, BMI, and presence of DM, CVD, hypertension exhibited significant differences between participants with and without trouble sleeping (*P* < .05). Moreover, compared with participants without trouble sleeping, those with trouble sleeping tended to be older, female, smoker, with a marital status of widowed/divorced/separated, a BMI ≥ 30 kg/m^2^, a caffeine intake ≥ 165 mg/d, and a presence of DM, CVD, and hypertension. Significantly, participants with trouble sleeping tended to possess higher urine tungsten, cobalt, and cadmium levels, but lower urine arsenic levels. By contrast, urine thallium, cesium, antimony, and molybdenum levels were not significantly different between 2 groups.

**Table 1:** Basic characteristics of participants based on sleep status.

Characteristic	Overall N = 10,012*	No-trouble sleeping N = 7477*	Trouble sleeping N = 2535*	*P*-value**
Age, yr				**<.001**
20–39 yr	3413 (34.1%)	2790 (37.3%)	623 (24.6%)	
40–59 yr	3314 (33.1%)	2329 (31.1%)	985 (38.9%)	
≥60 yr	3285 (32.8%)	2358 (31.5%)	927 (36.6%)	
Gender				**<.001**
Male	5026 (50.2%)	3958 (52.9%)	1068 (42.1%)	
Female	4986 (49.8%)	3519 (47.1%)	1467 (57.9%)	
Race/ethnicity				**<.001**
Mexican American	1496 (14.9%)	1230 (16.5%)	266 (10.5%)	
Other Hispanic	933 (9.3%)	724 (9.7%)	209 (8.2%)	
Non-Hispanic White	4417 (44.1%)	3061 (40.9%)	1356 (53.5%)	
Non-Hispanic Black	2127 (21.2%)	1619 (21.7%)	508 (20.0%)	
Other Race – Including Multiracial	1039 (10.4%)	843 (11.3%)	196 (7.7%)	
Education				**<.001**
<9th grade	994 (9.9%)	795 (10.6%)	199 (7.9%)	
9–11th grade (includes 12th grade with no diploma)	1404 (14.0%)	1071 (14.3%)	333 (13.1%)	
High school graduate/GED or equivalent	2314 (23.1%)	1705 (22.8%)	609 (24.0%)	
Some college or AA degree	2987 (29.8%)	2135 (28.6%)	852 (33.6%)	
College graduate or above	2313 (23.1%)	1771 (23.7%)	542 (21.4%)	
PIR	2.13 (1.12, 4.04)	2.14 (1.14, 4.01)	2.04 (1.07, 4.10)	.200
Marital status				**<.001**
Married/living with partner	6040 (60.3%)	4628 (61.9%)	1412 (55.7%)	
Never married	1784 (17.8%)	1394 (18.6%)	390 (15.4%)	
Widowed/divorced/separated	2188 (21.9%)	1455 (19.5%)	733 (28.9%)	
BMI				**<.001**
<25.0 kg/m^2^	2932 (29.3%)	2308 (30.9%)	624 (24.6%)	
25–29.9 kg/m^2^	3313 (33.1%)	2568 (34.3%)	745 (29.4%)	
≥30.0 kg/m^2^	3767 (37.6%)	2601 (34.8%)	1166 (46.0%)	
Smoking status	4565 (45.6%)	3203 (42.8%)	1362 (53.7%)	**<.001**
Caffeine intake				**<.001**
≤42 mg/d	3354 (33.5%)	2561 (34.3%)	793 (31.3%)	
43–164 mg/d	3323 (33.2%)	2513 (33.6%)	810 (32.0%)	
≥165 mg/d	3335 (33.3%)	2403 (32.1%)	932 (36.8%)	
DM	1282 (12.8%)	808 (10.8%)	474 (18.7%)	**<.001**
CVD	948 (9.5%)	563 (7.5%)	385 (15.2%)	**<.001**
Hypertension	3515 (35.1%)	2306 (30.8%)	1209 (47.7%)	**<.001**
Tungsten (μg/g Creatinine)	0.07 (0.04, 0.12)	0.07 (0.04, 0.12)	0.07 (0.04, 0.12)	**.015**
Arsenic (μg/g Creatinine)	7.24 (4.16, 15.12)	7.38 (4.26, 15.17)	6.74 (3.90, 14.95)	**<.001**
Thallium (μg/g Creatinine)	0.15 (0.11, 0.21)	0.15 (0.11, 0.21)	0.15 (0.10, 0.21)	.200
Cesium (μg/g Creatinine)	4.21 (3.09, 5.90)	4.22 (3.11, 5.85)	4.20 (3.05, 6.04)	>.900
Antimony (μg/g Creatinine)	0.05 (0.04, 0.08)	0.05 (0.04, 0.08)	0.05 (0.04, 0.08)	.500
Molybdenum (μg/g Creatinine)	38.66 (25.93, 57.56)	38.80 (26.18, 57.89)	38.14 (25.20, 56.97)	.100
Cobalt (μg/g Creatinine)	0.35 (0.24, 0.53)	0.34 (0.23, 0.52)	0.37 (0.24, 0.57)	**<.001**
Cadmium (μg/g Creatinine)	0.24 (0.13, 0.43)	0.22 (0.12, 0.42)	0.27 (0.15, 0.49)	**<.001**

Bold values denote statistical significance at the .05 level (*P* < .05).

BMI = body mass index, CVD = cardiovascular disease, DM = diabetes mellitus, PIR = poverty income ratio.

* n (%); Median (IQR).

** Pearson Chi-squared test; Wilcoxon rank sum test.

### 3.2. Association of single metal with trouble sleeping

**Figure 2.**
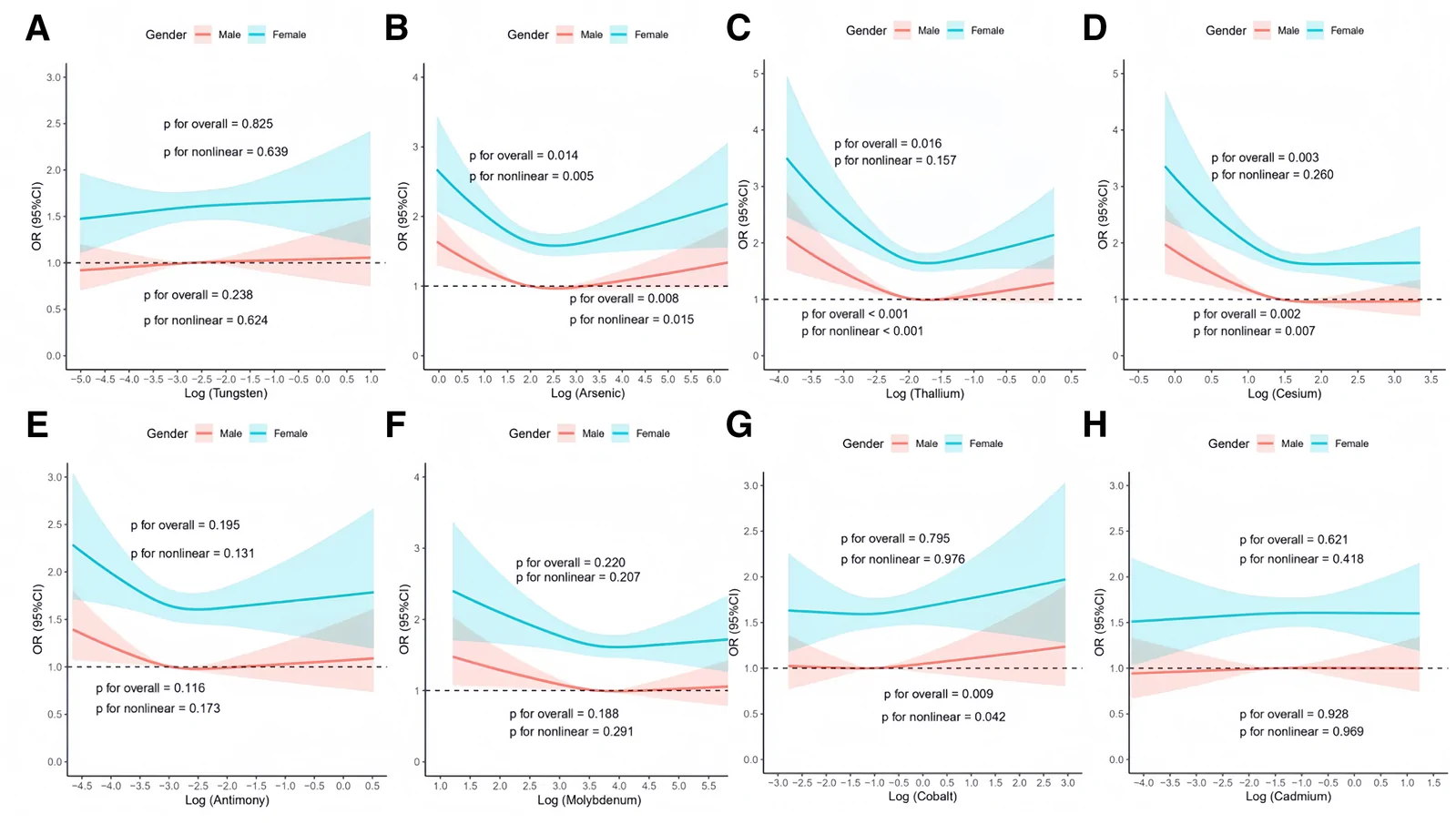
Restricted cubic spline analysis for the association between urine tungsten (A), arsenic (B), thallium (C), cesium (D), antimony (E), molybdenum (F), cobalt (G), cadmiumlevels (H) levels and trouble sleeping among males (red) and females (blue). RCS regression was adjusted for age, race, education level, PIR, marital status, BMI, smoking status, caffeine intake, DM, CVD, and hypertension. BMI = body mass index, CVD = cardiovascular disease, DM = diabetes mellitus, PIR = poverty income ratio, RCS = restricted cubic spline.

To analyze the association between urine metal levels and trouble sleeping, 2 weighted logistic regression models were established (Table 2), among which subgroup analysis according to gender were further performed to explore potential gender difference. Furthermore, RCS analyses were conducted to explore the dose–response relationship between urine metal levels and the risk of trouble sleeping (Figs. 2 and S1, Supplemental Digital Content 5).

**Table 2:** Association between 8 urinary metals and trouble sleeping.

Group	Crude model			Adjusted model		
	OR (95% CI)	*P*	*P* for trend	OR (95% CI)	*P*	*P* for trend
Tungsten						
Overall						
Q1			**.017**			.475
Q2	1.12 (0.99–1.28)	.078		1.09 (0.95–1.24)	.223	
Q3	1.18 (1.04–1.34)	**.012**		1.07 (0.94–1.23)	.297	
Q4	1.16 (1.02–1.32)	**.023**		1.06 (0.92–1.21)	.408	
Male						
Q1			**.024**			.073
Q2	1.33 (1.09–1.62)	**.004**		1.28 (1.05–1.57)	**.016**	
Q3	1.38 (1.13–1.67)	**.001**		1.30 (1.06–1.59)	**.012**	
Q4	1.26 (1.04–1.54)	**.021**		1.22 (0.99–1.50)	.056	
Female						
Q1			.790			.589
Q2	1.02 (0.86–1.21)	.817		0.99 (0.82–1.18)	.876	
Q3	1.06 (0.89–1.26)	.499		0.99 (0.83–1.19)	.923	
Q4	1.01 (0.85–1.20)	.895		0.95 (0.79–1.14)	.556	
Arsenic						
Overall						
Q1			**.002**			**.011**
Q2	0.84 (0.75–0.96)	**.008**		0.85 (0.75–0.97)	**.018**	
Q3	0.75 (0.66–0.85)	**<.001**		0.74 (0.64–0.85)	**<.001**	
Q4	0.85 (0.75–0.96)	**.009**		0.87 (0.76–1.00)	.050	
Male						
Q1			**.003**			**.022**
Q2	0.74 (0.62–0.90)	**.002**		0.74 (0.61–0.90)	**.003**	
Q3	0.75 (0.62–0.91)	**.003**		0.75 (0.62–0.92)	**.005**	
Q4	0.75 (0.62–0.90)	**.002**		0.78 (0.64–0.96)	**.017**	
Female						
Q1			.067			.424
Q2	0.89 (0.75–1.06)	.192		0.92 (0.77–1.10)	.370	
Q3	0.83 (0.70–0.99)	**.033**		0.87 (0.72–1.04)	.123	
Q4	0.87 (0.73–1.03)	.100		0.94 (0.78–1.14)	.544	
Thallium						
Overall						
Q1			.371			.073
Q2	0.82 (0.72–0.93)	**.002**		0.85 (0.74–0.97)	**.015**	
Q3	0.89 (0.79–1.01)	.083		0.88 (0.77–1.01)	.068	
Q4	0.92 (0.81–1.04)	.168		0.86 (0.75–0.99)	**.038**	
Male						
Q1			**.017**			.089
Q2	0.80 (0.67–0.97)	**.021**		0.83 (0.68–1.00)	.056	
Q3	0.70 (0.58–0.84)	**<.001**		0.74 (0.60–0.91)	**.003**	
Q4	0.83 (0.69–1.00)	**.046**		0.87 (0.71–1.06)	.163	
Female						
Q1			**.007**			.105
Q2	0.76 (0.64–0.90)	**.001**		0.79 (0.66–0.95)	**.011**	
Q3	0.82 (0.70–0.98)	**.025**		0.89 (0.74–1.06)	.187	
Q4	0.76 (0.64–0.90)	**.002**		0.82 (0.68–0.99)	**.038**	
Cesium						
Overall						
Q1			.992			**.001**
Q2	0.92 (0.81–1.04)	.195		0.87 (0.76–0.99)	**.037**	
Q3	0.87 (0.77–0.99)	**.034**		0.73 (0.63–0.84)	**<.001**	
Q4	1.02 (0.90–1.15)	.798		0.78 (0.68–0.91)	**.001**	
Male						
Q1			.110			**.007**
Q2	0.80 (0.66–0.97)	**.022**		0.79 (0.65–0.97)	**.022**	
Q3	0.82 (0.68–0.99)	**.036**		0.76 (0.62–0.94)	**.010**	
Q4	0.85 (0.70–1.02)	.087		0.74 (0.59–0.92)	**.006**	
Female						
Q1			.116			**.015**
Q2	0.93 (0.79–1.10)	.416		0.90 (0.75–1.09)	.281	
Q3	0.84 (0.71–1.00)	.050		0.77 (0.64–0.94)	**.008**	
Q4	0.90 (0.75–1.06)	.206		0.81 (0.67–0.99)	**.037**	
Antimony						
Overall						
Q1			.393			.219
Q2	0.96 (0.84–1.09)	.509		0.91 (0.79–1.04)	.148	
Q3	0.96 (0.85–1.09)	.569		0.88 (0.77–1.00)	.056	
Q4	1.06 (0.93–1.20)	.384		0.92 (0.81–1.06)	.246	
Male						
Q1			.327			.179
Q2	1.01 (0.83–1.22)	.936		0.99 (0.81–1.20)	.911	
Q3	0.84 (0.69–1.02)	.080		0.79 (0.65–0.97)	**.026**	
Q4	0.96 (0.79–1.16)	.671		0.93 (0.76–1.14)	.473	
Female						
Q1			.924			.258
Q2	0.86 (0.72–1.02)	.085		0.85 (0.71–1.02)	.080	
Q3	1.01 (0.85–1.19)	.935		0.97 (0.81–1.16)	.728	
Q4	0.96 (0.81–1.14)	.621		0.86 (0.72–1.03)	.097	
Molybdenum						
Overall						
Q1			.102			.086
Q2	0.92 (0.81–1.05)	.220		0.93 (0.81–1.06)	.263	
Q3	0.92 (0.81–1.05)	.220		0.91 (0.80–1.04)	.184	
Q4	0.89 (0.79–1.02)	.086		0.89 (0.77–1.02)	.082	
Male						
Q1			.084			.131
Q2	0.89 (0.74–1.08)	.234		0.91 (0.74–1.10)	.326	
Q3	0.86 (0.71–1.04)	.125		0.88 (0.72–1.07)	.198	
Q4	0.85 (0.70–1.03)	.090		0.86 (0.70–1.05)	.139	
Female						
Q1			.067			.424
Q2	0.89 (0.75–1.06)	.192		0.92 (0.77–1.10)	.370	
Q3	0.83 (0.70–0.99)	**.033**		0.87 (0.72–1.04)	.123	
Q4	0.87 (0.73–1.03)	.100		0.94 (0.78–1.14)	.544	
Cobalt						
Overall						
Q1			**<.001**			.586
Q2	0.96 (0.84–1.09)	.526		0.90 (0.78–1.04)	.141	
Q3	1.13 (0.99–1.28)	.067		0.95 (0.83–1.10)	.497	
Q4	1.29 (1.13–1.46)	**<.001**		1.02 (0.88–1.18)	.806	
Male						
Q1			.003			.122
Q2	0.95 (0.78–1.16)	.624		0.95 (0.77–1.17)	.629	
Q3	0.98 (0.81–1.19)	.850		0.96 (0.78–1.18)	.688	
Q4	1.33 (1.10–1.60)	**.003**		1.17 (0.96–1.44)	.126	
Female						
Q1			.260			.739
Q2	1.00 (0.84–1.19)	.990		1.06 (0.89–1.27)	.515	
Q3	0.94 (0.80–1.12)	.519		0.99 (0.82–1.19)	.881	
Q4	0.92 (0.77–1.09)	.333		0.99 (0.82–1.19)	.931	
Cadmium						
Overall						
Q1			**<.001**			.956
Q2	1.24 (1.08–1.41)	**.002**		1.00 (0.86–1.15)	.970	
Q3	1.46 (1.28–1.66)	**<.001**		0.98 (0.84–1.14)	.800	
Q4	1.71 (1.50–1.95)	**<.001**		1.01 (0.85–1.19)	.919	
Male						
Q1			**<.001**			.620
Q2	1.02 (0.84–1.25)	.831		0.92 (0.75–1.15)	.471	
Q3	1.26 (1.04–1.53)	**.018**		1.04 (0.83–1.31)	.730	
Q4	1.38 (1.14–1.67)	**.001**		1.03 (0.80–1.32)	.839	
Female						
Q1			**<.001**			.716
Q2	1.35 (1.13–1.62)	**<.001**		1.06 (0.87–1.28)	.577	
Q3	1.39 (1.17–1.67)	**<.001**		0.97 (0.79–1.18)	.729	
Q4	1.66 (1.39–1.98)	**<.001**		0.99 (0.80–1.23)	.935	

Bold values denote statistical significance at the .05 level (*P* < .05).

Crude model: unadjusted. Adjusted model: adjusted for gender (for overall), age, race/ethnicity, education, PIR, marital status, smoking status, caffeine intake, BMI, DM, CVD, and hypertension.

BMI = body mass index, CI = confidence interval, CVD = cardiovascular disease, DM = diabetes mellitus, OR = odds ratio, PIR = poverty income ratio.

As illustrated in Table 2, a higher urine tungsten level was found to be associated with an increased risk of trouble sleeping, yet no significant associations were observed in the fully adjusted model. We then revealed that a potential gender difference exists: a higher urine tungsten level was cross-sectionally associated with an increased risk of trouble sleeping in males but not females. After adjustment of potential confounders, ORs (95% CIs) for trouble sleeping in the second and third quartiles of urine tungsten were 1.28 (1.05–1.57) and 1.30 (1.06–1.59) compared to the lowest quartile (Q1), respectively. Dramatically, a negative association was found between urine arsenic and trouble sleeping for all participants and males (*P* for trend < .05). In males, the adjusted ORs (95% CIs) in the increasing quartiles of urine arsenic were 0.74 (0.61–0.90), 0.75 (0.62–0.92), and 0.78 (0.64–0.96) compared to the Q1 group, respectively. For urine thallium, the Q1 group showed a statistical association with the highest risk of trouble sleeping for all participants in both models. In males, a significantly lower risk was found in the Q3 of urine tungsten after adjustment. Females exhibited a consistent significant reduction in risk across all quartiles in the crude model, with some attenuation in the adjusted model but still significant in the Q2 and Q4 groups. Similarly, a negative association has been observed between urine cesium and trouble sleeping (*P* for trend < .05). The adjusted ORs (95% CIs) in the increasing quartiles of urine cesium were 0.87 (0.76–0.99), 0.73 (0.63–0.84), and 0.78 (0.68–0.91) compared to the Q1, respectively. Additionally, RCS curve displayed negative and nonlinear associations between urine arsenic, thallium, cesium levels and trouble sleeping (*P* for overall < .05, *P* for nonlinearity < .05), which varied in gender groups (Figs. 2 and S1, Supplemental Digital Content 5).

As described in Table 2, no significant association was observed for urine antimony and molybdenum levels with trouble sleeping among all participants (*P* > .05). However, in males but not females, an antimony level in the Q3 group was observed to be associated with a lower risk of trouble sleeping compared to the Q1 group (OR: 0.79, 95% CI: 0.65–0.97). For urine cobalt and cadmium, significantly positive associations were observed between levels of these 2 metals and trouble sleeping in the crude model. However, after fully adjusted for all confounders, these associations became insignificant (*P* > .05). Subsequently, RCS curve displayed that no significant nonlinearity was found for association of urine antimony, molybdenum, cobalt, cadmium with trouble sleeping (*P* for nonlinearity > .05; Figs. 2 and S1, Supplemental Digital Content 5).

### 3.3. Association of mixed metals with trouble sleeping

To further explore the combined effects of multiple metals, WQS regression models were applied to assess the joint association of metal mixtures with trouble sleeping, stratified by gender. Prior to constructing the WQS model, the correlations among the concentrations of metals were evaluated. The results indicated that most metals exhibited low to moderate correlations, with no evidence of severe multicollinearity, supporting their inclusion as a mixture in the WQS analysis (Fig. S2, Supplemental Digital Content 1).

**Figure 3.**
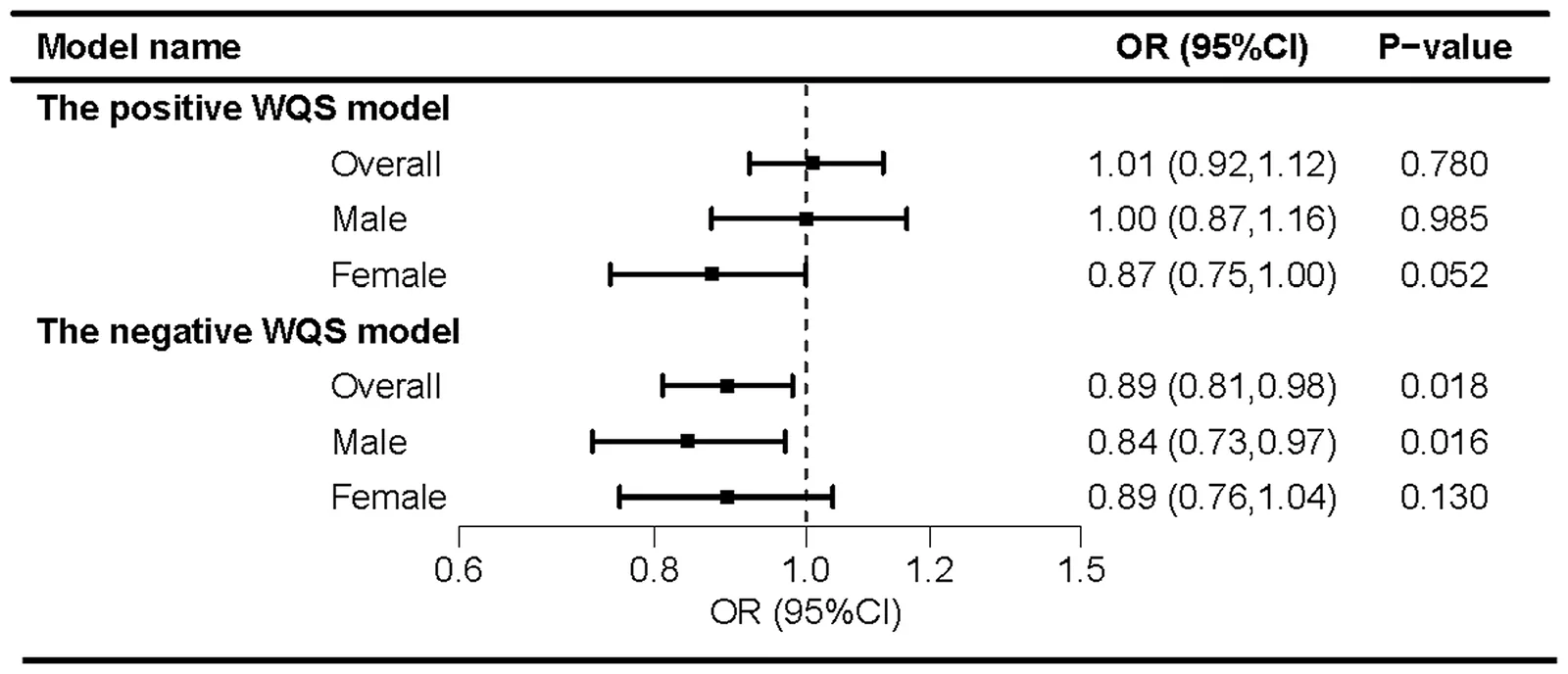
Association between urinary metals and trouble sleeping, by WQS model. Models were adjusted for age, gender (for overall), race, education level, PIR, marital status, BMI, smoking status, caffeine intake, DM, CVD, and hypertension. BMI = body mass index, CI = confidence interval, CVD = cardiovascular disease, DM = diabetes mellitus, OR = odds ratio, PIR = poverty income ratio, WQS = weighted quantile sum.

In WQS analysis of the total population, the positive model did not show a significant association (OR = 1.01, 95% CI: 0.92–1.12; Fig. 3), despite that tungsten exhibited a relatively high weight (Fig. S3, Supplemental Digital Content 2). Conversely, the negative model showed a significant adjusted inverse association (OR = 0.89, 95% CI: 0.81–0.98), dominated by cesium and arsenic, aligning with their robust dose-dependent protection in single-metal analyses. For males, the positive model revealed no adjusted association (OR = 1.00, 95% CI: 0.87–1.16), despite crude trends linked to cobalt and cadmium. In contrast, the negative model demonstrated a clear inverse association (OR = 0.84, 95% CI: 0.73–0.97), driven by arsenic and cesium. In females, the positive model after adjustment suggested a marginal protective trend (OR = 0.87, 95% CI: 0.75–1.00), while the negative model showed no significance (OR = 0.89, 95% CI: 0.76–1.04).

### 3.4. Sensitivity analysis

To assess the robustness of our findings, we conducted a sensitivity analysis focusing on the influence of extreme values. As shown in Figure S4, Supplemental Digital Content 3, several extreme values were identified in the distribution of urinary metal concentrations. After outlier adjustment, the results of the logistic regression remained consistent with those from the primary analysis (Table S1, Supplemental Digital Content 4), with the direction and magnitude of associations largely unchanged. Notably, the inverse associations for arsenic, cesium, and thallium with trouble sleeping persisted and remained statistically significant. Similarly, since winsorization of outliers does not affect quantile rankings, the results of the WQS regression in the sensitivity analysis remained consistent with the primary findings as indicated in Figure 3.

## 4. Discussion

In this comprehensive analysis of data from the NHANES 2005–2018, we examined the associations between the urinary concentrations of 8 metals (tungsten, arsenic, thallium, cesium, antimony, molybdenum, cobalt, and cadmium) and trouble sleeping, revealing significant but varied relationships.

We found a possible positive association between urinary tungsten and trouble sleeping in males, with the WQS analysis providing some support for this finding, as tungsten showed a prominent weight in the positive model, although it did not reach statistical significance. To our knowledge, this is the first study revealing this correlation. This positive association may be explained by tungsten potential to induce oxidative stress and disrupt normal biological processes.^[22,23]^ Given the emerging evidence of tungsten potential health impacts, particularly its role in oxidative stress, it is important to further investigate its broader implications for health.

The observed male-specific positive association between urinary tungsten levels and trouble sleeping may reflect sex-related differences in metal toxicokinetics and endocrine interactions. Prior studies indicate that toxic metals are processed differently in males and females due to variations in absorption, distribution, metabolism, and excretion. For example, men may metabolize certain metals less efficiently, leading to higher internal exposure and more pronounced health effects.^[24,25]^ In addition, many metals are considered endocrine-disrupting chemicals that may interfere with melatonin synthesis and circadian regulation. Such hormonal disruption has been shown to vary by sex, suggesting differential neuroendocrine responses in males and females.^[26]^ These mechanisms may underlie the sex-specific finding. Future research including sex-stratified toxicokinetic assessments and hormonal pathway analyses are needed to clarify these interactions.

Using both single and mixed metal models, we observed that higher urinary arsenic, cesium, and thallium were unexpectedly associated with lower risk of trouble sleeping. Several explanations may account for this. First, reverse causation is possible – individuals with poor sleep may alter behaviors (e.g., diet and supplement use), unintentionally affecting metal exposure or excretion. Second, unmeasured confounders (e.g., environmental or lifestyle factors) might bias the associations. Biologically, low-level exposure to certain toxicants (e.g., arsenic) may trigger adaptive stress responses or hormesis, potentially influencing sleep regulation. Rahman et al^[14]^ reported inverse associations between specific arsenic species and sleep disorders. However, another NHANES study linked arsenic to leg jerks, but not nighttime awakenings,^[13]^ suggesting outcome-specific effects. For cesium, while Shiue found no association between urinary cesium levels and sleep health,^[13]^ Lestaevel et al^[17]^ observed altered sleep patterns in animals exposed to ^137^Cesium, indicating possible isotope or dose-dependent effects. Thallium inverse link may reflect individual susceptibility or adaptive responses. Overall, these findings underscore the complex, nonlinear nature of metal-sleep interactions and warrant further investigation.

By contrast, no significant associations were found for antimony and molybdenum with trouble sleeping after adjusting for confounders. For antimony, a study based on NHANES 2005–2006 found positive associations between urinary antimony levels and insufficient sleep as well as obstructive sleep apnea (OSA).^[19]^ However, another study using NHANES 2009–2016 data found no significant associations.^[18]^ Our data, spanning from 2005 to 2018, also found no significant links between urinary antimony levels and trouble sleeping, suggesting that the effects of antimony on sleep may be minimal or influenced by other factors not captured in these studies. For molybdenum, previous research has suggested that its urinary concentration is not significantly associated with leg cramps during sleep,^[13]^ which is consistent with our findings. The lack of significant associations suggests that, if these metals do impact sleep health, the effects might be subtle or mediated through complex pathways that are not directly captured by urinary measurements alone.

Regarding cobalt and cadmium, both metals showed positive associations with trouble sleeping in the crude model, but these associations became insignificant after full adjustment. For cobalt, one previous research based on general populations found that blood cobalt levels were significantly higher in OSA patients compared to non-OSA groups, but this was based on univariate analysis.^[16]^ In fact, 2 other studies suggested that urinary cobalt concentration is not significantly associated with sleep disorders,^[13,20]^ which aligns with our conclusions. For cadmium, previous research findings have been mixed: some studies found that high blood cadmium levels were positively associated with the risk of sleep disorders or OSA,^[10,11]^ while others reported no significant associations with sleep duration or leg cramps during sleep.^[12,13]^ This inconsistency indicates the need for more comprehensive studies to understand the true impact of cadmium on sleep health.

Overall, our findings suggested that monitoring and regulating environmental exposures to certain metals could be an important consideration in addressing sleep health issues. Given the significant associations observed, particularly with tungsten, public health interventions aimed at reducing exposure to this metal may help alleviate some sleep disturbances in the population. Furthermore, the gender differences observed indicate that personalized approaches may be necessary when considering the impact of environmental exposures on sleep health. The results from the WQS analysis, which revealed the combined effects of multiple metals, further emphasized the complexity of these relationships and underscored the need for a more holistic approach to understanding the environmental factors influencing sleep health.

Our study has notable strengths, one of which is the large, nationally representative sample from NHANES 2005–2018, enhancing the robustness and generalizability of our findings. Notably, our sample encompasses a racially/ethnically diverse population (Mexican American, other Hispanic, non‑Hispanic White, non-Hispanic Black, and other Race-Including Multiracial), and all analyses were adjusted for these categories. Besides, we expanded the scope to include several metals that have been less studied in the context of sleep health, thus addressing a significant gap in the existing literature. However, our study has several limitations. First, the cross-sectional nature of the data precludes any causal inference. Longitudinal studies or experimental designs are needed to establish causality and explore underlying mechanisms. Second, trouble sleeping was assessed using a single, self-reported binary item (SLQ050), which – despite its common use in NHANES studies – is a crude measure subject to recall bias. It lacks detail on sleep duration, quality, and specific disorders. Future studies should incorporate validated instruments such as the Pittsburgh Sleep Quality Index or Insomnia Severity Index to improve measurement precision. Third, urinary metal concentrations reflect short-term exposure and may not accurately capture long-term exposure, limiting inference on chronic effects. Biomarkers of long-term exposure, such as hair, nails, or repeated urine measurements, may better capture chronic exposure effects.

## 5. Conclusion

In conclusion, our study suggested that higher urinary tungsten levels were possibly linked to increased risk of trouble sleeping in males, whereas arsenic, thallium, and cesium were cross-sectionally associated with decreased risk of trouble sleeping, with notable gender differences. No significant associations were found for antimony, molybdenum, cobalt, and cadmium. Longitudinal research is needed to confirm these associations, elucidate the underlying biological mechanisms, and assess the combined effects of multiple metal exposures on sleep health.

## Author contributions

**Data curation:** Qiong Ma, Jian-Zheng Liu, Li-Li Li, Xiao-Wei Feng, Mi Fu, Jian-Min Lv.

**Formal analysis:** Qiong Ma, Jian-Zheng Liu, Li-Li Li, Xiao-Wei Feng, Xiang Fan, Mi Fu, Jian-Min Lv.

**Funding acquisition:** Mi Fu, Jian-Min Lv.

**Methodology:** Qiong Ma, Li-Li Li, Xiao-Wei Feng, Xiang Fan.

**Project administration:** Jian-Zheng Liu, Xiu-E. Shi, Mi Fu, Jian-Min Lv.

**Supervision:** Jian-Zheng Liu, Xiu-E. Shi, Mi Fu, Jian-Min Lv.

**Writing – original draft:** Qiong Ma.

**Writing – review & editing:** Qiong Ma, Jian-Zheng Liu, Xiu-E. Shi, Li-Li Li, Xiao-Wei Feng, Xiang Fan, Mi Fu, Jian-Min Lv.










