# Supplementary material for: Association of eight urinary metals and trouble sleeping: A cross-sectional study
Source: Medicine (Baltimore). 2026 Sep 4;105(36):e50551. doi: 10.1097/MD.0000000000050551 (PMC13549585; doi:10.1097/MD.0000000000050551)
Supplement: Supplementary file 1 [file medi-105-e50551-s004.doc]

**Table S1 Association between eight urinary metals and trouble sleeping (extreme values excluded)**

|  | **Group** | **Crude model** | | |  | **Adjusted model** | | |
| --- | --- | --- | --- | --- | --- | --- | --- | --- |
|  |  | **OR (95%CI)** | ***p*** | ***p* for trend** |  | **OR (95%CI)** | ***p*** | ***p* for trend** |
| **Tungsten** |  |  |  |  |  |  |  |  |
| Overall | Q1 | **Ref** |  | 0.055 |  |  |  | 0.761 |
|  | Q2 | 1.11 (0.99-1.26) | 0.084 |  |  | 1.07 (0.94-1.21) | 0.308 |  |
|  | Q3 | 1.12 (0.99-1.27) | 0.079 |  |  | 1.03 (0.90-1.18) | 0.667 |  |
|  | Q4 | 1.13 (1.00-1.28) | 0.059 |  |  | 1.03 (0.90-1.17) | 0.667 |  |
| Male | Q1 | **Ref** |  | 0.111 |  |  |  | 0.210 |
|  | Q2 | 1.24 (1.04-1.48) | **0.017** |  |  | 1.23 (1.03-1.48) | **0.026** |  |
|  | Q3 | 1.12 (0.93-1.36) | 0.234 |  |  | 1.07 (0.87-1.30) | 0.531 |  |
|  | Q4 | 1.19 (0.98-1.44) | 0.074 |  |  | 1.18 (0.96-1.44) | 0.108 |  |
| Female | Q1 | **Ref** |  | 0.959 |  |  |  | 0.435 |
|  | Q2 | 0.98 (0.82-1.16) | 0.784 |  |  | 0.93 (0.78-1.10) | 0.389 |  |
|  | Q3 | 1.04 (0.87-1.23) | 0.67 |  |  | 0.97 (0.81-1.17) | 0.768 |  |
|  | Q4 | 0.99 (0.83-1.17) | 0.87 |  |  | 0.91 (0.77-1.09) | 0.325 |  |
| **Arsenic** |  |  |  |  |  |  |  |  |
| Overall | Q1 | **Ref** |  | **0.002** |  |  |  | **0.017** |
|  | Q2 | 0.85 (0.75-0.96) | **0.009** |  |  | 0.86 (0.76-0.99) | **0.030** |  |
|  | Q3 | 0.75 (0.66-0.85) | **<0.001** |  |  | 0.75 (0.66-0.86) | **<0.001** |  |
|  | Q4 | 0.85 (0.75-0.96) | **0.010** |  |  | 0.88 (0.77-1.01) | 0.068 |  |
| Male | Q1 | **Ref** |  | **0.006** |  |  |  | **0.044** |
|  | Q2 | 0.79 (0.66-0.95) | **0.012** |  |  | 0.81 (0.67-0.98) | **0.028** |  |
|  | Q3 | 0.75 (0.62-0.91) | **0.003** |  |  | 0.78 (0.64-0.95) | **0.013** |  |
|  | Q4 | 0.78 (0.65-0.95) | **0.011** |  |  | 0.83 (0.67-1.01) | 0.065 |  |
| Female | Q1 | **Ref** |  | **0.009** |  |  |  | 0.149 |
|  | Q2 | 0.86 (0.72-1.02) | 0.086 |  |  | 0.92 (0.77-1.11) | 0.396 |  |
|  | Q3 | 0.70 (0.58-0.83) | **<0.001** |  |  | 0.74 (0.61-0.89) | **0.002** |  |
|  | Q4 | 0.83 (0.70-0.99) | **0.035** |  |  | 0.92 (0.77-1.11) | 0.396 |  |
| **Thallium** |  |  |  |  |  |  |  |  |
| Overall | Q1 | **Ref** |  | 0.468 |  |  |  | 0.182 |
|  | Q2 | 0.79 (0.70-0.90) | **<0.001** |  |  | 0.81 (0.71-0.92) | **0.002** |  |
|  | Q3 | 0.94 (0.83-1.06) | 0.302 |  |  | 0.93 (0.81-1.05) | 0.245 |  |
|  | Q4 | 0.93 (0.82-1.04) | 0.209 |  |  | 0.88 (0.77-1.01) | 0.073 |  |
| Male | Q1 | **Ref** |  | 0.253 |  |  |  | 0.765 |
|  | Q2 | 0.78 (0.65-0.93) | **0.007** |  |  | 0.84 (0.69-1.01) | 0.058 |  |
|  | Q3 | 0.87 (0.73-1.05) | 0.155 |  |  | 0.94 (0.77-1.14) | 0.518 |  |
|  | Q4 | 0.91 (0.75-1.11) | 0.369 |  |  | 0.98 (0.79-1.21) | 0.85 |  |
| Female | Q1 | **Ref** |  | **0.004** |  |  |  | 0.154 |
|  | Q2 | 0.73 (0.60-0.87) | **<0.001** |  |  | 0.77 (0.64-0.94) | **0.01** |  |
|  | Q3 | 0.82 (0.69-0.97) | **0.023** |  |  | 0.90 (0.75-1.08) | 0.269 |  |
|  | Q4 | 0.74 (0.63-0.87) | **<0.001** |  |  | 0.83 (0.69-0.99) | **0.041** |  |
| **Cesium** |  |  |  |  |  |  |  |  |
| Overall | Q1 | **Ref** |  | 0.972 |  |  |  | <0.001 |
|  | Q2 | 0.92 (0.81-1.04) | 0.179 |  |  | 0.87 (0.76-0.99) | **0.039** |  |
|  | Q3 | 0.87 (0.76-0.99) | **0.030** |  |  | 0.73 (0.64-0.85) | **<0.001** |  |
|  | Q4 | 1.02 (0.90-1.15) | 0.813 |  |  | 0.81 (0.70-0.93) | **0.004** |  |
| Male | Q1 | **Ref** |  | 0.265 |  |  |  | 0.029 |
|  | Q2 | 0.84 (0.71-1.00) | 0.053 |  |  | 0.83 (0.69-1.00) | **0.046** |  |
|  | Q3 | 0.79 (0.65-0.95) | **0.014** |  |  | 0.74 (0.60-0.90) | **0.004** |  |
|  | Q4 | 0.95 (0.78-1.16) | 0.635 |  |  | 0.83 (0.66-1.03) | 0.096 |  |
| Female | Q1 | **Ref** |  | 0.064 |  |  |  | **0.010** |
|  | Q2 | 0.92 (0.76-1.11) | 0.396 |  |  | 0.94 (0.77-1.15) | 0.542 |  |
|  | Q3 | 0.79 (0.66-0.95) | **0.014** |  |  | 0.75 (0.62-0.92) | **0.005** |  |
|  | Q4 | 0.86 (0.72-1.03) | 0.107 |  |  | 0.80 (0.65-0.98) | **0.032** |  |
| **Antimony** |  |  |  |  |  |  |  |  |
| Overall | Q1 | **Ref** |  | 0.355 |  |  |  | 0.325 |
|  | Q2 | 0.87 (0.75-1.00) | 0.056 |  |  | 0.83 (0.71-0.96) | **0.013** |  |
|  | Q3 | 1.05 (0.93-1.17) | 0.435 |  |  | 0.97 (0.86-1.09) | 0.582 |  |
|  | Q4 | 1.03 (0.92-1.16) | 0.596 |  |  | 0.92 (0.81-1.05) | 0.208 |  |
| Male | Q1 | **Ref** |  | 0.479 |  |  |  | 0.311 |
|  | Q2 | 0.74 (0.59-0.93) | **0.008** |  |  | 0.71 (0.56-0.89) | **0.004** |  |
|  | Q3 | 0.93 (0.78-1.11) | 0.41 |  |  | 0.88 (0.73-1.05) | 0.165 |  |
|  | Q4 | 0.94 (0.79-1.13) | 0.533 |  |  | 0.93 (0.77-1.13) | 0.454 |  |
| Female | Q1 | **Ref** |  | 0.590 |  |  |  | 0.063 |
|  | Q2 | 0.93 (0.77-1.14) | 0.496 |  |  | 0.92 (0.75-1.13) | 0.429 |  |
|  | Q3 | 1.07 (0.91-1.25) | 0.397 |  |  | 1.04 (0.88-1.22) | 0.67 |  |
|  | Q4 | 1.01 (0.87-1.19) | 0.856 |  |  | 0.93 (0.79-1.10) | 0.392 |  |
| **Molybdenum** |  |  |  |  |  |  |  |  |
| Overall | Q1 | **Ref** |  | 0.106 |  |  |  | 0.060 |
|  | Q2 | 0.92 (0.81-1.05) | 0.220 |  |  | 0.92 (0.81-1.05) | 0.212 |  |
|  | Q3 | 0.93 (0.82-1.05) | 0.230 |  |  | 0.91 (0.79-1.04) | 0.151 |  |
|  | Q4 | 0.90 (0.79-1.02) | 0.088 |  |  | 0.87 (0.76-1.00) | 0.055 |  |
| Male | Q1 | **Ref** |  | 0.117 |  |  |  | 0.142 |
|  | Q2 | 0.86 (0.72-1.03) | 0.106 |  |  | 0.87 (0.72-1.05) | 0.151 |  |
|  | Q3 | 0.92 (0.76-1.10) | 0.358 |  |  | 0.91 (0.75-1.10) | 0.334 |  |
|  | Q4 | 0.84 (0.69-1.02) | 0.071 |  |  | 0.84 (0.69-1.04) | 0.104 |  |
| Female | Q1 | **Ref** |  | **0.028** |  |  |  | 0.235 |
|  | Q2 | 0.94 (0.79-1.13) | 0.521 |  |  | 0.97 (0.80-1.16) | 0.713 |  |
|  | Q3 | 0.87 (0.73-1.04) | 0.125 |  |  | 0.90 (0.75-1.09) | 0.291 |  |
|  | Q4 | 0.84 (0.70-0.99) | **0.042** |  |  | 0.91 (0.75-1.09) | 0.297 |  |
| **Cobalt** |  |  |  |  |  |  |  |  |
| Overall | Q1 | **Ref** |  | **<0.001** |  |  |  | 0.428 |
|  | Q2 | 0.95 (0.84-1.08) | 0.467 |  |  | 0.90 (0.79-1.04) | 0.143 |  |
|  | Q3 | 1.14 (1.00-1.29) | **0.045** |  |  | 0.98 (0.85-1.12) | 0.745 |  |
|  | Q4 | 1.29 (1.14-1.46) | **<0.001** |  |  | 1.03 (0.90-1.19) | 0.633 |  |
| Male | Q1 | **Ref** |  | **<0.001** |  |  |  | 0.060 |
|  | Q2 | 0.86 (0.72-1.02) | 0.076 |  |  | 0.85 (0.71-1.02) | 0.079 |  |
|  | Q3 | 1.00 (0.83-1.21) | 0.969 |  |  | 0.94 (0.77-1.15) | 0.556 |  |
|  | Q4 | 1.62 (1.32-1.99) | **<0.001** |  |  | 1.35 (1.08-1.69) | **0.009** |  |
| Female | Q1 | **Ref** |  | 0.175 |  |  |  | 0.648 |
|  | Q2 | 0.92 (0.75-1.13) | 0.45 |  |  | 0.95 (0.77-1.18) | 0.663 |  |
|  | Q3 | 0.95 (0.79-1.15) | 0.614 |  |  | 1.00 (0.82-1.23) | 0.988 |  |
|  | Q4 | 0.87 (0.73-1.05) | 0.147 |  |  | 0.94 (0.77-1.15) | 0.561 |  |
| **Cadmium** |  |  |  |  |  |  |  |  |
| Overall | Q1 | **Ref** |  | **<0.001** |  |  |  | 0.254 |
|  | Q2 | 1.29 (1.13-1.47) | **<0.001** |  |  | 1.10 (0.95-1.27) | 0.195 |  |
|  | Q3 | 1.48 (1.29-1.68) | **<0.001** |  |  | 1.07 (0.92-1.25) | 0.380 |  |
|  | Q4 | 1.71 (1.51-1.94) | **<0.001** |  |  | 1.12 (0.95-1.32) | 0.173 |  |
| Male | Q1 | **Ref** |  | **<0.001** |  |  |  | 0.769 |
|  | Q2 | 1.14 (0.95-1.36) | 0.148 |  |  | 1.04 (0.86-1.27) | 0.664 |  |
|  | Q3 | 1.23 (1.02-1.49) | **0.034** |  |  | 1.00 (0.79-1.26) | 0.988 |  |
|  | Q4 | 1.38 (1.14-1.67) | **0.001** |  |  | 1.06 (0.82-1.36) | 0.669 |  |
| Female | Q1 | **Ref** |  | **<0.001** |  |  |  | 0.189 |
|  | Q2 | 1.38 (1.13-1.69) | **0.002** |  |  | 1.23 (0.99-1.51) | 0.058 |  |
|  | Q3 | 1.54 (1.27-1.87) | **<0.001** |  |  | 1.20 (0.97-1.49) | 0.088 |  |
|  | Q4 | 1.76 (1.46-2.13) | **<0.001** |  |  | 1.22 (0.97-1.53) | 0.088 |  |

Crude model: unadjusted. Adjusted model: adjusted for gender (for overall), age, race/ethnicity, education, PIR, marital status, smoking status, caffeine intake, BMI, DM, CVD, and hypertension. Extreme values were excluded in this analysis.
